# Preparation of amphiphilic magnetic polyvinyl alcohol targeted drug carrier and drug delivery research

**DOI:** 10.1080/15685551.2020.1837442

**Published:** 2020-10-23

**Authors:** Yazhen Wang, Zhen Shi, Yu Sun, Xueying Wu, Shuang Li, Shaobo Dong, Tianyu Lan

**Affiliations:** a College of Materials Science and Engineering, Qiqihar University, Qiqihar, China; b Heilongjiang Provincial Key Laboratory of Polymeric Composite Materials, Qiqihar, China; c College of Chemistry, Chemical Engineering and Resource Utilization, Northeast Forestry University, Harbin, China

**Keywords:** Fe_3_O_4_, magnetic, Poly(vinyl alcohol), targeted drug delivery, amphiphilic

## Abstract

Currently, magnetic applications have great potential for development in the field of drug carriers. In this paper, Fe_3_O_4_-PVA@SH, an amphiphilic magnetically targeting drug carrier, was prepared by using Fe_3_O_4_ and PVA with thiohydrazide-iminopropyltriethoxysilane(TIPTS). The loading capacity of Fe_3_O_4_-PVA@SH on Aspirin and the drug release kinetics of loaded drugs were studied. The obtained Fe_3_O_4_-PVA@SH exhibits excellent drug release properties in simulating the human body fluid environment (pH 7.2). Since magnetically targeting drug carriers are readily available and have excellent biocompatibility and the characteristics of drug release. This work’s development, preparing amphiphilic magnetically targeting drug carriers in drug delivery and other fields, has great significance.

## 1. Introduction

Nanotechnology helps develop new pharmaceutical agents, drug delivery, and the synthesis of drug carriers [1–3]. Playing a vital role in treating human diseases (such as malignant tumors and heart disease) by using magnetic cores to target therapeutic drugs. [4–7] Recently, efforts include targeted delivery. Drugs are only active in specific areas of the body (such as cancerous areas or lesions), and medications can be released in a controlled manner over a while [8–12]. Magnetic nanoparticles are a carrier form used for targeted therapy, with a particle size between 1 ~ 100 nm. The magnetic nanoparticles concentrate the drug carrier in the target region through the magnetic field. The drug can be released smoothly, increase the target’s concentration, enhance the therapeutic effect, reduce the distribution in other parts, and reduce toxicity and side effects. The drug carrier controls the release of the drug to have an excellent therapeutic effect. The loading and releasing of drugs must be performed [13–16].The core part of the magnetically targeting drug carrier is iron oxide nanoparticles due to the superparamagnetic and single domain characteristics of iron oxide nanoparticles [17–21].The target is provided by a magnetic polymer made of Fe_3_O_4_ as a magnetic core and is coated with the magnetic polymer. The polymer’s purpose is to make Fe_3_O_4_ nanoparticles as a magnetic core to be better and more uniformly distributed in the drug carrier. When Fe_3_O_4_ nanoparticles are coated with high molecular polymers, they can be used in drug carriers. Superparamagnetic iron oxide was used extensively in the detection of atherosclerotic plaque. And expanded-pore nanoparticles functionalized with N-isopropyl acrylamide and poly(ethylene glycol) were applied for temperature control release of bovine hemoglobin. Compared with small molecule drugs with passive targeting, polymer-drug carriers generally exhibit better pharmacokinetics. [22–26]

**Table 1.** The magnetization of Fe_3_O_4_, Fe_3_O_4_-PVA, and Fe_3_O_4_-PVA@SH at 300 K

Samples	Magnetization(emu/g)
Fe_3_O_4_	0.78
Fe_3_O_4_-PVA	0.52
Fe_3_O_4_-PVA@SH	0.22

In the drug carrier’s actual application, the targeted drug carrier should have good biocompatibility and accurately target the body fluid environment’s desired location [27,28].As a magnetic material, Fe_3_O_4_ is widely used in human treatment because of its stable quality, superparamagnetic properties, and easy realization. Fe_3_O_4_ nanoparticles are widely used as biomaterials and exhibit superparamagnetic properties in magnetic resonance imaging (MRI), targeted hyperthermia, drug delivery, and immobilized proteins [29–33].However, as a magnetically targeting drug carrier, its biocompatibility is poor. A variety of natural and synthetic biodegradable polymers are used for drug delivery [34–36].Among various polymers, polyvinyl alcohol (PVA) has received more and more attention. It is a biodegradable, biocompatible, water-soluble, and inexpensive polymer [37–40].It has good water solubility, good film-forming properties, adhesion, emulsification, and good solvent resistance because PVA molecules have more hydroxyl groups [41–45].Pharmacological experiments have proved that PVA is non-toxic, tasteless, non-irritating to the skin, and will not cause skin allergies. It has been widely used as a drug carrier. When PVA is used in a drug carrier, it exhibits sustained-release properties due to its macromolecular swelling properties. Sustained-release drugs can reduce dosing frequency and improve patients’ compliance, especially children and elderly patients [46–49].

Suppose the magnetical drug carriers want to show excellent biocompatibility. In that case, it needs to improve the solubility of the water of their carriers and improve their solubility of lipids. The feature of this study is to improve the lipid solubility of drug carriers through thiohydrazide-iminopropyltriethoxysilane (TIPTS). TIPTS is not only playing a coupling role in the polymerization reaction but also improve the lipid solubility. Good biocompatibility requires good amphiphilic [50,51]. The thiolated polymer and the cysteine-rich(Cys) thiol group in the Cys subdomain of the mucosal glycoprotein form a disulfide bond has strong adhesion and good cohesion [52–56].

Aspirin (acetylsalicylic acid), with a chemical formula C_9_H_8_O_4_, is a widely used medicine. Aspirin is a cyclooxygenase (COX) inhibitor. It mainly reduces the synthesis of thromboxane (TXA2) by inhibiting COX activity, thus preventing platelet aggregation and blood clotting. [57–61] Aspirin is also used to prevent first heart attacks.

**Scheme 1.**
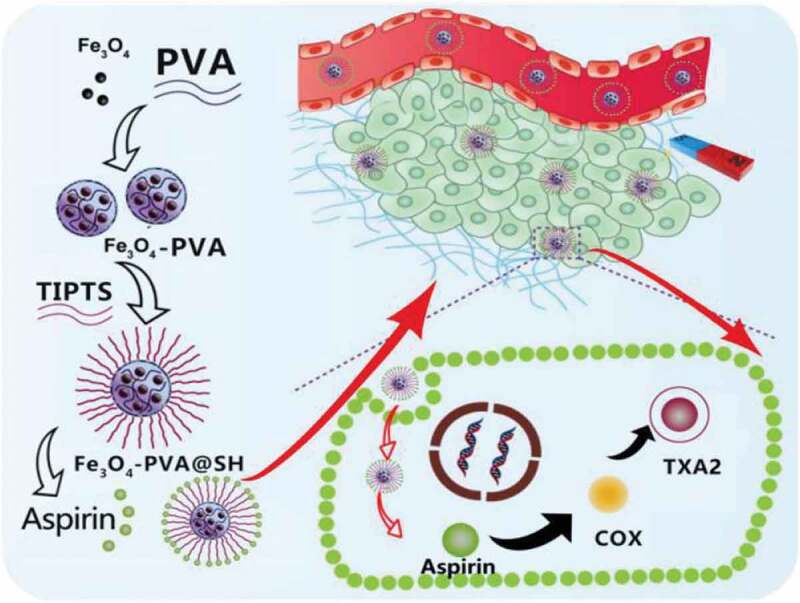
Schematic illustration of the synthetic route of Fe_3_O_4_-PVA@SH and the proposed synergistic antithrombotic mechanism of Fe_3_O_4_-PVA@SH

In this project, Fe_3_O_4_ is used as a magnetically targeted magnetic carrier. They were improving the water solubility of Fe_3_O_4_ by the reaction of Fe_3_O_4_ and PVA to get the Fe_3_O_4_-PVA. At the same time, the swelling characteristics of PVA makes the entire drug carrier exhibit slow-release characteristics. The Fe_3_O_4_-PVA reacts with thiohydrazide-aminopropyl-riethoxysilane (TIPTS) to synthesize Fe_3_O_4_-PVA @ SH improving the fat solubility of the drug carrier. On the other hand, the hydroxyl group on Aspirin can form a hydrogen bond(-OH) with the hydroxyl group(-OH) and thiol group(-SH) on Fe_3_O_4_-PVA @ SH and is loaded on the Fe_3_O_4_-PVA @ SH carrier, which is transported to the lesion site by the carrier and exerts efficacy. The mechanism is shown in scheme 1.

## 2. Experimental section

### 2.1. Materials

Poly(vinyl alcohol) (PVA) (Mw 4505 (degree of alcoholysis) 87.0–89.0 mol%, viscosity 80.0–110.0 mpa.s), Aspirin (acetylsalicylic acid), Iron(II) chloride tetrahydrate (FeCl_2_ · 4H_2_O), iron(III) chloride hexahydrate(FeCl_3_ · 6H_2_O), [55] ammonia solution (25 wt%) were purchased from Aladdin Chemistry Co. Ltd. (Shanghai, China). Dimethyl sulfoxide (DMSO) was purchased from Sinopharm Chemical Reagent Co., Ltd (Shanghai, China). Concentrated sulfuric acid (H_2_SO_4_) was purchased from Acros Organics (Beijing, China). thiohydrazide-iminopropyltrithoxy-silane (TIPTS) [55], homemade.

### 2.2. *Synthesis of magnetic (Fe_3_O_4_) nanoparticles* [62]

FeCl_3_ · 6H_2_O (4 g) and FeCl_2_ · 4H_2_O (2 g) were dissolved in 100 mL of distilled water. The solution ultrasound for an hour. Then, 30 mL of ammonia (NH_3_) was added to the solution and stirred for five hours at 70°C. The entire reaction system was carried out under nitrogen protection. Finally, the product was rinsed repeatedly, rinsed with water, and freeze-dried.

### 2.3. *Synthesis of Fe_3_O_4_-PVA* [62]

PVA (3 g) was dissolved in 100 mL of distilled water and stirred using a mechanical stirrer to dissolve. Fe_3_O_4_ (3 g) was ultrasound for an hour and add it in the PVA solution; the ammonia solution was added to adjust the pH appropriately. The entire reaction system was reacted for 5 h under the protection of nitrogen. The black product was washed with distilled water. Until the solution as the whole system reached a neutral pH, and the samples were freeze-dried.

### 2.4. Synthesis of Fe_3_O_4_-PVA@SH

**Figure 1.**
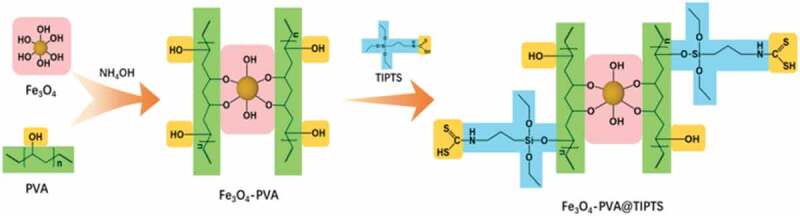
The synthetic route of Fe_3_O_4_-PVA@SH

The thiol coupling agent(4 g) and Fe_3_O_4_-PVA(1 g) were dispersed into 100 mL of DMSO. The solution ultrasound for 2 h and add H_2_SO_4_ until the solution system’s pH is 1 to 2.Then the entire reaction system was stirred at 35°C for 2 h. Also, the product was centrifuged, rinsed with water, and freeze-drying. At this point, we get Fe_3_O_4_-PVA@SH. The experimental process is shown in Figure 1.

### 2.5. Characterization

X-ray powder diffraction (XRD) spectra were taken on a Holland PANalytical X− Pert PRO X-ray diffractometer with Cu−Kα radiation. Fourier transforms infrared (FTIR) spectra were performed on the IRAffinity-1 spectrometer. Infrared spectrum analysis. Scanning electron microscopy (SEM)and Energy Dispersive Spectrometer (EDS) images were recorded using the JSM-6380 LV microscope. Contact angle measuring instrument (JC2000D1, Shanghai). Differential scanning calorimetry (DSC) was carried on a NETZSCH STA 449 C analyzer with a heating rate of 20 °C min^−1^ in nitrogen flow.

#### 2.5.1. Swelling measurements

The swelling properties of the Fe_3_O_4_-PVA@SH were determined. The swelling ratio was calculated as follows:

swellingratio=(Ws−Wd)/Wd

*Wd* and *Ws* are the weight of dried Fe_3_O_4_-PVA@SH before and after immersing in aqueous solution for 48 h, respectively.

#### 2.5.2. Loading kinetics studies

To investigate the loading kinetics of the Fe_3_O_4_-PVA@SH for Aspirin, we typically left 45 mg of Fe_3_O_4_-PVA@SH to soak in 10 mL of an aqueous solution of Aspirin(0.085 mmol/L) at 37°C temperature. After predetermined intervals time, the supernatant solution was collected for analysis by UV spectrophotometer. The amount of Aspirin loaded by Fe_3_O_4_-PVA@SH the was calculated from the following mass balance equation:

$$Qt=\left(Co-Ct\right)/m$$

*Qt* (mmol/g) is the amount adsorbed per gram of Fe_3_O_4_-PVA@SH at time *t, Co* is the initial concentration of Aspirin in the solution (mmol/L), *Ct* is the concentration of Aspirin at time *t* (mmol/L), *V* is the volume of the solution (L), and *m* is the mass of the Fe_3_O_4_-PVA@SH used(g).

#### 2.5.3. Drug release from Fe_3_O_4_-PVA@SH

For the drug release experiment, the release of Aspirin was determined with a UV−vis spectrophotometer at λmax = 287 nm at a function of time. The typical procedure used as follows: the above aspirin-loaded Fe_3_O_4_-PVA@SH were kept immersed in 3 mL water of pH = 7.2 at 37°Cand placed on a shaking machine a certain shaking frequency to simulate the process of drug release in the human body. At particular intervals, the supernatant solution was collected for analysis by a UV spectrophotometer. Each experiment was carried out in triplicate.

$$Release=\frac{theamountofaspirinreleasedfromFe_{3}O_{4}-\text{\textbackslash{}breakPVA}@SH\text{\textbackslash{}break}}{thetotalamountofaspirinloadedbyFe_{3}O_{4}-\text{\textbackslash{}breakPVA}@SH}\times 100\%$$

## 3. Results and discussion

### 3.1. XRD

**Figure 2.**
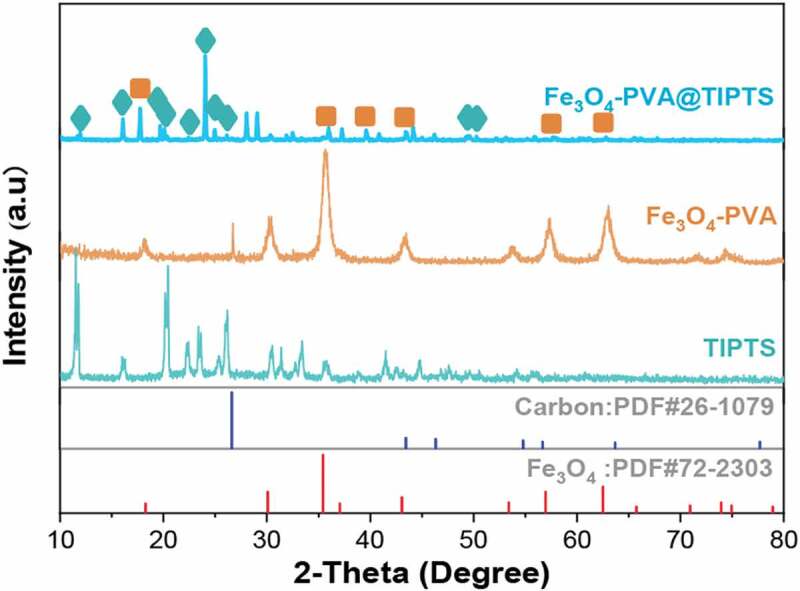
XRD curves of Fe_3_O_4_-PVA, TIPTS, and Fe_3_O_4_-PVA@SH

According to the XRD pattern, Figure 2 shows the XRD pattern of Fe_3_O_4_-PVA, TIPTS and Fe_3_O_4_-PVA@SH nanocomposite. Compared with standard cards. We can be seen that the peaks at 2θ = 30.1°, 35.5°, 43.3°, 57.3°, and 62.7° were assigned to the characteristic peaks of Fe_3_O_4_ (JCPDS card No. 72–2303), demonstrated that Fe_3_O_4_ particles were successfully formed in the PVA matrix. The peaks at 2θ = 26.6°, 43.4°, 54.8°, 56.6°, and 63.6° are given to the typical carbon peak of TIPTS(JCPDS card No.26–1097) and confirmed the PVA’s semicrystalline properties. The XRD patterns of Fe_3_O_4_ -PVA shows that the synthesis of Fe_3_O_4_-PVA was successful [63,64]. The XRD pattern of the nanoparticles containing Fe_3_O_4_-PVA@SH is shown in Figure 2. Comparing the characteristic peaks on the XRD curves of Fe_3_O_4_-PVA, TIPTS, and Fe_3_O_4_-PVA @ SH in the figure. Some characteristic peaks of TIPTS have partially deviated. This phenomenon is because that PVA and TIPTS are coated outside Fe_3_O_4_, which makes the particle size of Fe_3_O_4_ change massive, the crystal form changes slightly. All shows that PVA and TIPTS are added to the core structure, so this analysis’s composite construction is quite evident.

### 3.2. FT-IR analysis

**Figure 3.**
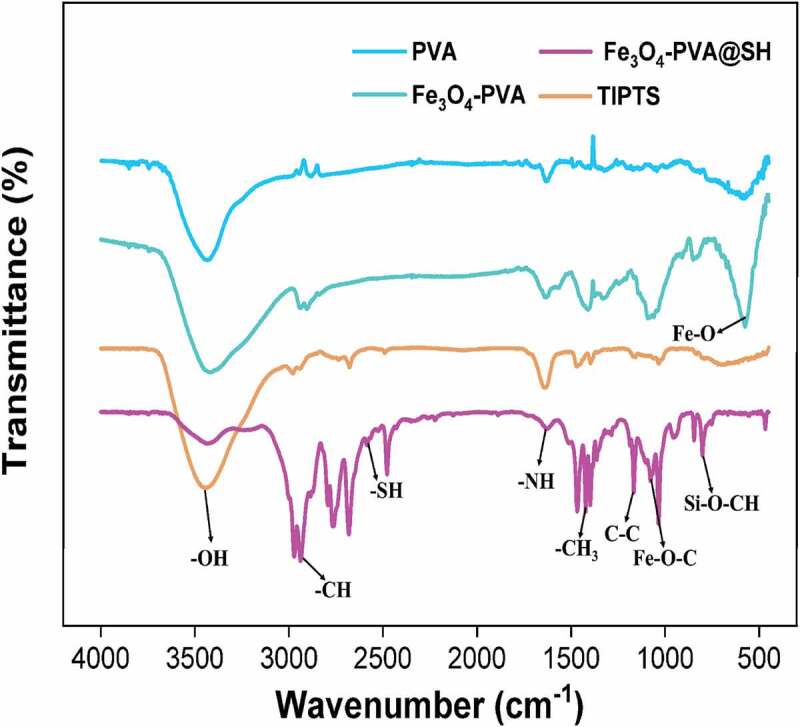
FT-IR spectrum of Fe_3_O_4_ and Fe_3_O_4_-PVA, TIPTS, and Fe_3_O_4_-PVA@SH

Infrared spectroscopy was performed to analyze the chemical changes between the incorporated components. The FT-IR spectra of PVA and Fe_3_O_4_-PVA are shown in Figure 3. The peak seen at 3439 cm^−1^ is attributable to the -OH stretching vibration of PVA. The peak at 2902 cm^−1^ in the infrared spectrum of Fe_3_O_4_-PVA is due to the stretching vibration of -CH. The peak at 1416 cm^−1^ is due to the stretching vibration of -C-C-, the rise at 1096 cm^−1^ is the stretching vibration peak of Fe-O-C, and the rise at 569 cm^−1^ is due to the stretching vibration of Fe-O- vibration peak. The existence of characteristic peaks indicates that we successfully synthesized Fe_3_O_4_-PVA. Figure 3 shows the infrared spectra of TIPTS and Fe_3_O_4_-PVA@SH. The mountain seen in TIPTS at 3435 cm^−1^ is due to the stretching vibration of -OH. The peak at 2583 cm^−1^ in Fe_3_O_4_-PVA @ SH is -SH, the height at 1432 cm^−1^ is caused by the hydrocarbon bending vibration of -CH_3_, and the stretching vibration of -CH causes the peak at 2928 cm^−1^. The height at 1373 cm^−1^ is the typical stretching vibration of -C-C-. The height at 1083 cm^−1^ is the stretching vibration peak of Fe-O-C. The height at 1628 cm^−1^ is the stretching vibration peak -NH, the peak at 798 cm^−1^ is attributed to the stretching vibration peak of Si-O-CH_3_. There is no stretching vibration of Fe-O, indicating that Fe_3_O_4_ is encapsulated to form a magnetic core drug carrier system Fe_3_O_4_ with thiols and hydroxyl groups. The synthesis of Fe_3_O_4_-PVA@SH was successful based on the synthesis of Fe-O-C, Si-O-CH_3,_ and the presence of -SH and -NH, which confirms the formation of Fe_3_O_4_-PVA@SH.

### 3.3. SEM and EDS

**Figure 4.**
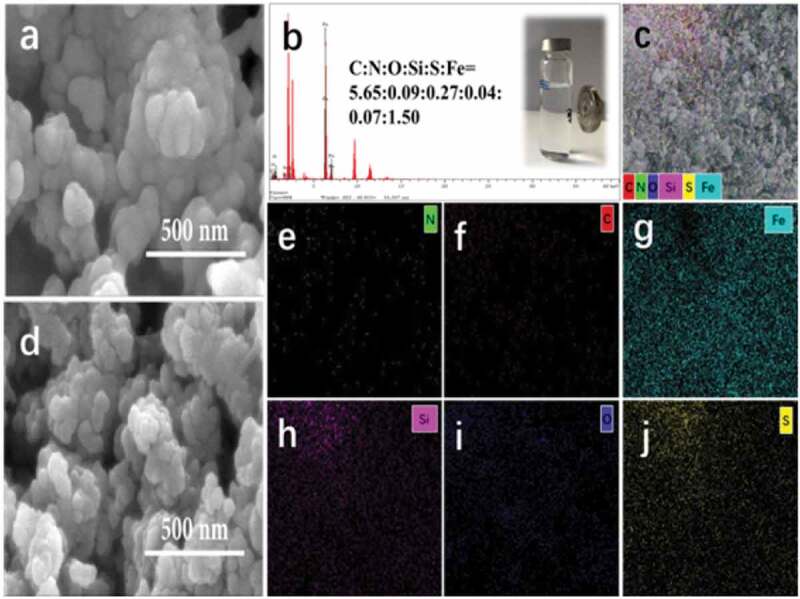
SEM images of (a)Fe_3_O_4_-PVA and of (d) Fe_3_O_4_-PVA@SH; (b) EDS images of Fe_3_O_4_-PVA@SH and Fe_3_O_4_-PVA@SH magnetic display; (c, e-j)EDS images of Fe_3_O_4_-PVA@SH

Figure 4(a) shows the SEM images of the Fe_3_O_4_-PVA synthesized. Accordingly, the synthesis of uniformly distributed spherical structures with a particle diameter of about 60 nm can be seen. Figure 4(d) shows the SEM images of nanoparticles containing Fe_3_O_4_-PVA @SH with a particle diameter of about 100 nm. Among them, the spherical irregularities are a mixture of PVA and TIPTS. Fe_3_O_4_ nanoparticles in which the polymeric that cover the core are well visible.

Energy dispersive spectra analysis was performed to the elemental composition of the nanoparticles and confirmed the product’s purity Figure 4(b) shows the EDS pattern of Fe_3_O_4_-PVA. The samples contain C, O, and Fe elements. The elemental analysis obtained from this spectrum indicates that C: O: Fe atomic ratio is 1:1.7:1.1. Figure 4(c) shows the EDS pattern of Fe_3_O_4_-PVA@SH the samples contain N(Figure 4(e)), C(Figure 4(f)), Fe(Figure 4(g)), Si(Figure 4(h)), O(Figure 4(i)), and S(Figure 4(j)) elements, and that the parts are evenly distributed on the sample.

### 3.4. Contact angle and nanoparticle size

**Figure 5.**
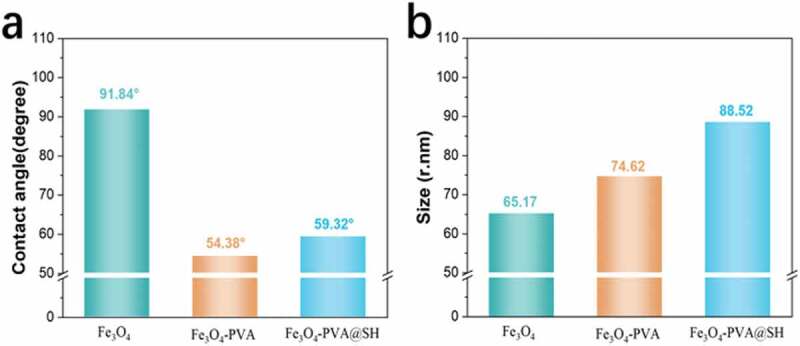
(a)The contact angle histogram shows the changing trend of the contact angle of Fe_3_O_4_, Fe_3_O_4_-PVA, and Fe_3_O_4_-PVA@SH;(b)The particle size histogram shows the changing direction of the contact angle of Fe_3_O_4_, Fe_3_O_4_-PVA and Fe_3_O_4_-PVA@SH

Figure 5(a) shows the contact angles of Fe_3_O_4,_ Fe_3_O_4_-PVA and Fe_3_O_4_-PVA@SH are 91.84 degrees, 54.38 degrees, and 59.32 degrees. Figure 5(b) shows that the particle sizes of Fe_3_O_4_, Fe_3_O_4_-PVA, and Fe_3_O_4_-PVA@SH are 65.17 nm, 74.62 nm, and 88.52 nm, respectively. It has been well documented that the size of less than 100 nm is favorable for passive targeting. Fe_3_O_4_ is coated with polyvinyl alcohol, making Fe_3_O_4_-PVA have an increased particle size and increased hydrophilicity compared to Fe_3_O_4_. And Fe_3_O_4_-PVA@SH is grafted with TIPTS outside, so the particle size of Fe_3_O_4_-PVA@SH is also slightly increased compared to Fe_3_O_4_-PVA. And improve the lipid solubility, so the corresponding hydrophilicity has been reduced. Nanoparticle size change again proves the formation of magnetically targeted drug carrier Fe_3_O_4_-PVA@SH. Respectively, Fe_3_O_4_ has some hydroxyl groups on the surface, but the number is too small to meet the drug carrier’s hydrophilicity requirements. PVA is a hydrophilic polymer. PVA has strong hydrophilicity. PVA has many hydroxyl groups and is coated on Fe_3_O_4_ to improve the carrier’s hydrophilicity. The mercapto group (-SH) is less water-soluble than the hydroxyl group (-OH), so the presence of the mercapto group (-SH) makes the carrier’s water solubility slightly lower. At the same time, a suitable drug carrier also needs to have excellent lipophilicity. The thiol group on TIPTS has strong nucleophilicity. The introduction of the thiol group improves the lipid solubility. Improving amphiphilicity allows magnetic targeting drug carriers to have excellent hydrophilicity and lipophilicity, and biological activity is much improved. Moreover, The magnetic targeting drug carrier can form a hydrogen-bonded co-loading drug with the drug, pass through the layers of cells, and target the drug to be transported under the action of an external magnetic field to exert the drug effect and improve the bioavailability.

### 3.5. VSM

**Figure 6.**
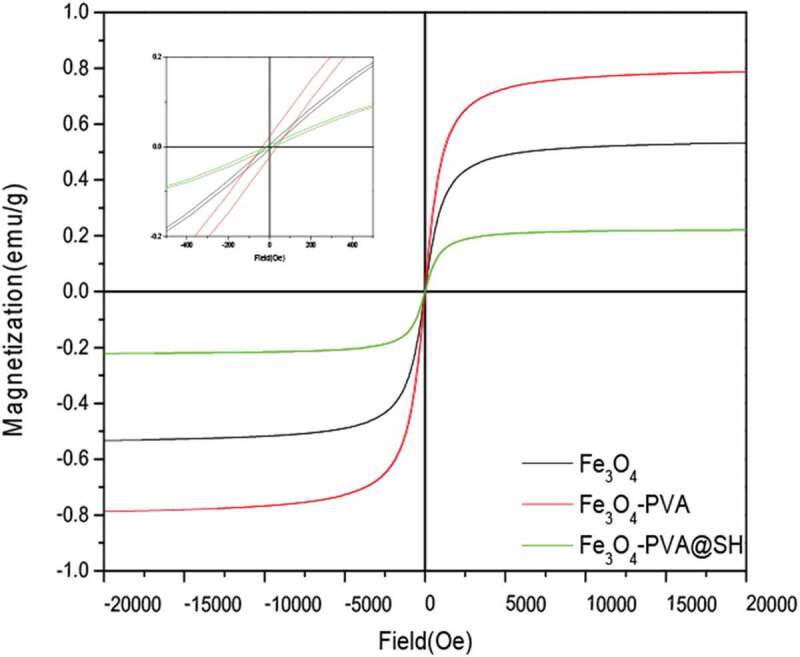
Magnetic hysteresis loops of the Fe_3_O_4_, Fe_3_O_4_-PVA, and Fe_3_O_4_-PVA@SH at 300 K

(The upper left is the detailed picture).

Meanwhile, the magnetic properties of Fe_3_O_4_, Fe_3_O_4_-PVA, and Fe_3_O_4_-PVA@SH were measured by VSM at 300 K (Figure 6) and Table 1. The hysteresis loop shows that Fe_3_O_4_, Fe_3_O_4_-PVA, and Fe_3_O_4_-PVA@SH were superparamagnetic with no coercivity at room temperature. The saturation magnetization values for Fe_3_O_4_, Fe_3_O_4_-PVA, and Fe_3_O_4_-PVA@SH are 0.78emu/g, 0.52emu/g, and 0.22 emu/g, respectively, which means that PVA and TIPTS are wrapped around Fe_3_O_4_, which further explains Fe_3_O_4_-PVA@SH preparation was successful. Superparamagnetism of drug carriers is very important for practical applications because, under a particular magnetic field, the drug release performance of Fe_3_O_4_-PVA@SH may be seriously affected by its magnetic strength [65].

### 3.6. DSC

**Figure 7.**
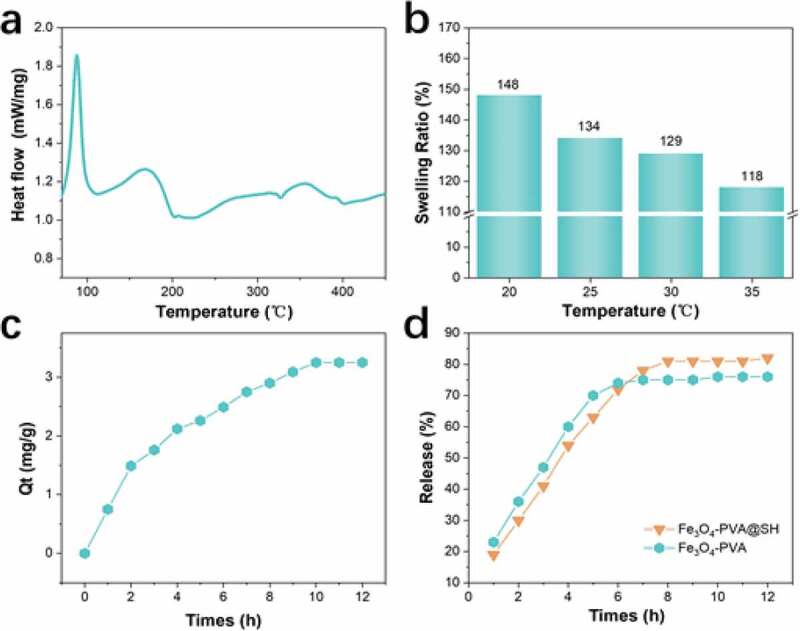
(a) DSC curves of Fe_3_O_4_-PVA@SH; (b) swelling ratio of Fe_3_O_4_-PVA@SH synthesized at 20 °C, 25 °C, 30 °Cand35°C; (c) effect of contact time on the and loading amount of Aspirin (0.085 mmol/L, 10 mL) by Fe_3_O_4_-PVA@SH (45 mg) at 37°C when pH = 7.2; (d) drug release profiles of Fe_3_O_4_-PVA and Fe_3_O_4_-PVA@SH at pH 7.2 and 37 temperature

Various ingredients of the nanoparticles were also characterized by differential Scanning Calorimeter (DSC) in Figure 7(a) DSC was performed from 60 °C to 450 °C in the N_2_ atmosphere with a heating rate of 10°C/min. A degree of about 60 °C to 100 °C there is related to water (moisture) evaporation. At around 100°C to 200 °C, there is a loss regarding side -OH elimination and TIPTS defunctionalization reactions decomposition and 200°C to 330 °C C-C cleavage chain rupture and is decomposed. At a temperature higher than 330°C, residual carbon and Fe_3_O_4_ has remained.

### 3.7. Swelling ratio

Also, the swelling ratio of the Fe_3_O_4_-PVA@SH synthesized at 20°C, 25°C, 30°C, and 35°C was studied, as shown in Figure 7(b). The swelling rates of Fe_3_O_4_-PVA @ SH synthesized at 20°C, 25°C, 30°C, and 35°C were 148%, 134%, 129%, and 118%, respectively. Also, the stability of the Fe_3_O_4_-PVA@SH was studied. First, the Fe_3_O_4_-PVA@SH were immersed in an aqueous solution for 48 h, and then the Fe_3_O_4_-PVA@SH were separated, and the aqueous residue solution was evaporated and weighed. It is found that all the Fe_3_O_4_-PVA@SH showed tiny (below 5 wt %) weight loss.

It is well known that the molecular chain structure of the PVA determines the swelling rate, and the reaction temperature can affect the void structure between the molecular chains, thereby affecting the swelling ratio. It can be seen from Figure 7(b) that the swelling ratio is best at 20, and the void structure is more.

### 3.8. Loading kinetics studies

For drug delivery applications, the drug carrier’s loading level is a crucial parameter in practical applications. Here, we choose Aspirin as a model drug to study the loading drug characteristics of the magnetically targeted drug carrier by Fe_3_O_4_-PVA@SH. The dry Fe_3_O_4_-PVA@SH was immersed in the aspirin solution for 12 hours to carry out the process of loading aspirin. As shown in Figure 7(c), the loading level of Fe_3_O_4_-PVA@SH is mainly because the PVA chain can adsorb aspirin molecules through strong interactions (such as van der Waals interactions and hydrogen bonds). There are a lot of sulfhydryls (-SH) groups on TIPTS. Disulfide bonds will be formed between the sulfhydryl polar groups at the end of TIPTS and dispersed between the pores of Fe_3_O_4_-PVA@SH. For better detection, the drug loading process and drug loading kinetics of Fe_3_O_4_-PVA @ SH were studied. The effect of contact time on Fe_3_O_4_-PVA@SH loaded aspirin is shown in Figure 7(c). The initial concentration of Aspirin is 0.085 mmol/L. It can be seen that 50% of Aspirin was loaded in 4 hours, and the sample loading process reached equilibrium in about 10 hours (Figure 7c). Correspondingly, the loading amount of Aspirin on Fe_3_O_4_-PVA@SH was 2.11 mg/g at 4 h and 3.21 mg/g at 10 h (Figure 7c). We believe that the fast loading rate in the first 4 h is mainly due to Aspirin’s adsorption on the outermost layer of Fe_3_O_4_-PVA@SH. After the outer layer reaches the load balance, the inside of Fe_3_O_4_-PVA@SH starts to slow down Aspirin’s adsorption. Finally, the internal and external adsorption equilibrium is reached.

### 3.9. Drug release kinetics studies

Because the Fe_3_O_4_-PVA@SH can load a large number of drugs, it is convenient to investigate their drug release properties in vitro. To investigate the TIPTS study of targeted drug carriers, we compared the drug release profiles of Fe_3_O_4_-PVA. Two samples, including Fe_3_O_4_-PVA and Fe_3_O_4_-PAV@SH chosen and their drug release profiles over time, were presented in Figure 7(d), as observed, significant differences in drug release rates and amount between the two samples. Within 4 h, the release rates of Fe_3_O_4_-PVA and Fe_3_O_4_-PVA @ SH are faster, and the release amount of Fe_3_O_4_-PVA reaches 60% at 4 h, while Fe_3_O_4_-PAV@ SH is 54%. This phenomenon may be explained as follows: This is due to the rapid release of Aspirin released by the drug carrier’s outer layer through strong interactions (van der Waals interaction and hydrogen bonding). The release rate of Fe_3_O_4_-PVA was gentle at 4 h to 6 h, which was due to the slow release of Aspirin adsorbed by the internal pores of PVA swelling. After 6 h, the release rate of Fe_3_O_4_-PVA is almost zero, and the release behavior stops. It can be seen that the drug release rate of Fe_3_O_4_-PVA @ SH has a slow-release process after 6 h and continues to 8 h. We think that the disulfide bond is formed between the thiol groups of TIPTS, and the disulfide bond break requires more energy than the hydrogen group, which slows the release of Aspirin and makes Fe_3_O_4_-PVA @SH reach a 3 h slower than Fe_3_O_4_-PVA release time.

PVA acts as a matrix, and -OH on the PVA chain can form strong interactions with drugs (van der Waals interaction and hydrogen bonding). At the same time, magnetic iron oxide nanoparticles provide targeting for the carrier. TIPTS is used as a biomaterial to improve the lipid solubility of magnetic polyvinyl alcohol. The thiol group on TIPTS can form a disulfide bond internally. It can also include a disulfide bond with the cysteine-rich(Cys) sulfhydryl group in the Cys subdomain of the cell surface mucosal glycoprotein. Well, the auxiliary PVA shows a better sustained-release effect. Fe_3_O_4_-PVA @SH showed high drug loading levels during the experiment. At the same time, drug release experiments showed that the drug release rate and quantity of Fe_3_O_4_-PVA@SH reached the release requirements of the 2019 US Pharmacopoeia Aspirin sustained-release tablets. We prepared a targeted magnetically drug carrier with a high potential for drug delivery and prevented the whole body’s excess distribution and eliminated its side effects. Our magnetic systems can easily reach the target point by applying an external magnetic field, while the superparamagnetic requires a smaller area than previously published works. When these nanoparticles begin to decompose inside the body, soluble iron is harmful and can be used in the patient with iron-deficiency anemia.

## 4. Conclusions

In conclusion, we successfully proved that the coupling agent (TIPTS) could be used in biology as a material for improving amphiphilicity and improving the liposolubility of magnetic drugs. Aspirin can be administered orally by loading on a magnetically targeting nanocarrier. The magnetic targeting drug carrier prepared experimentally has an excellent drug loading rate and a stable release for 8 hours. The present work is of interest for opening up enormous opportunities to make full use of magnetic carrier material in drug delivery and other applications, because of their easy availability, cost-effective productivity, and profitable drug release performance.
